# Data Transmission Reduction in Wireless Sensor Network for Spatial Event Detection

**DOI:** 10.3390/s21217256

**Published:** 2021-10-31

**Authors:** Marcin Lewandowski, Bartłomiej Płaczek

**Affiliations:** Institute of Computer Science, University of Silesia, Będzińska 39, 41-200 Sosnowiec, Poland; marcin.lewandowski@us.edu.pl

**Keywords:** wireless sensor network, event detection, lifetime, energy consumption, transmission reduction, cargo monitoring

## Abstract

Wireless sensor networks have found many applications in detecting events such as security threats, natural hazards, or technical malfunctions. An essential requirement for event detection systems is the long lifetime of battery-powered sensor nodes. This paper introduces a new method for prolonging the wireless sensor network’s lifetime by reducing data transmissions between neighboring sensor nodes that cooperate in event detection. The proposed method allows sensor nodes to decide whether they need to exchange sensor readings for correctly detecting events. The sensor node takes into account the detection algorithm and verifies whether its current sensor readings can impact the event detection performed by another node. The data are transmitted only when they are found to be necessary for event detection. The proposed method was implemented in a wireless sensor network to detect the instability of cargo boxes during transportation. Experimental evaluation confirmed that the proposed method significantly extends the network lifetime and ensures the accurate detection of events. It was also shown that the introduced method is more effective in reducing data transmissions than the state-of-the-art event-triggered transmission and dual prediction algorithms.

## 1. Introduction

Wireless sensor networks (WSNs) are composed of spatially distributed sensor nodes with sensing, data processing, and communication capabilities. The combination of these capabilities allows WSN to detect events and unusual behaviors in a monitored environment. Event detection is an important application area of WSNs which has gained significant attention in recent years [1,2]. There has been growing interest in the potential use of WSNs for the detection of military targets, physical security threats, natural hazards, technical malfunctions, etc. [3,4,5,6].

Events are usually defined as deviations from normal observations in the temporal or spatial domain. Temporal events can be detected as abrupt changes in data readings of a single sensor node. In contrast, spatial events are recognized by taking into account the sensor readings of neighboring nodes [7]. The spatial events correspond to spatial anomalies that occur in single points or small regions, where the values of monitored attributes are significantly inconsistent with those of their neighborhoods [8]. Thus, the sensor nodes in WSN have to exchange collected data in order to detect spatial events. The examples of such events include the presence of a malfunctioning device emitting pollution [9], contaminant intrusion to a water distribution system [10,11], oil leak from a pipeline [12], and stability loss of container during transportation [13].

One of the essential requirements for successful event detection applications is the long lifetime of WSN [14]. The lifetime of battery-powered wireless sensor nodes strongly depends on the number of data transmissions. Since wireless transmission is the most energy-consuming operation in WSN, the lifetime of sensor nodes can be significantly extended by reducing the data communication [15]. Thus, for event detection applications, methods are being sought to enable the accurate detection of events in real time and savings of energy resources by eliminating unnecessary transmissions.

In the literature, various methods have been presented to date to reduce data transmissions in wireless sensor networks. However, the available techniques—which are based on data aggregation [16], compression [17], prediction [18], or adaptive sampling [19]—do not take into account the particular algorithm which is used to detect the events in a given application. Thus, those methods cannot effectively eliminate data transmissions that are not necessary for a given algorithm to correctly recognize the events of interest. In the context of event detection, some dedicated data reduction methods were proposed for simple threshold-based event detection algorithms [20]. Such methods are not suitable when the threshold value is not explicitly defined. This means that their applicability is low when the event detection in WSNs is implemented with the use of machine learning algorithms. It should also be noted here that the event detection approach was used in the literature to reduce data transmissions in WSN. According to that approach, the reduction in transmissions is achieved as a sensor node sends data to a sink node only when an event of interest is triggered at the sensor node [1]. Such event-triggered methods assume that events have to be detected by a single sensor node—based on its own sensor readings. This approach is not relevant to detecting spatial events, based on data collected from neighboring sensor nodes.

The method presented in this paper was devised for the data transmission reduction in applications where data from neighboring sensor nodes have to be collected for detecting spatial events. In the considered WSN, events have been detected by the parent sensor node, which receives data from child sensor nodes. The parent node needs its own sensor readings and child node readings to correctly recognize the events. The proposed method assumes that the child node decides whether its current sensor readings must be transmitted to the parent node. To this end, the child node takes into account the event detection algorithm and verifies whether its current sensor readings can impact the results of event recognition performed by the parent node based on previously transmitted data. Specifically, the child node checks whether the result of event detection based on previously transmitted data may be different to the event detection result obtained with the current data. If the impact of current sensor readings on the event detection result is possible, then the child node sends the new data to the parent node. This approach allows the child node to skip the transmissions of data that are not necessary for the parent node to correctly recognize the events.

The proposed transmission reduction method was implemented in a WSN for cargo monitoring during transportation. The considered WSN detects the instability of cargo boxes in a vehicle based on data collected from accelerometers and gyroscopes. This system is composed of a parent sensor node attached to the vehicle body and child sensor nodes inside the cargo boxes. The parent node detects events related to movements and tilts of the cargo boxes inside a car by taking into account the measurements of vibrations and angular velocities made for the cargo boxes and the body of the vehicle. This WSN was used as a testbed for the experimental assessment of the introduced transmission reduction method in a real-world scenario. During the experiments, the ability to extend the lifetime of the sensor nodes and the impact on the event detection accuracy of the proposed method were compared with those of state-of-the-art approaches.

The main contributions of this work are summarized as follows:

- A new method is presented for data transmission reduction in WSN where spatial events have to be detected based on data from neighboring sensor nodes. The method is based on predicting the possible errors of event detection that can be encountered when current sensor readings are not reported;
- The introduced method was implemented for event detection in a cargo monitoring system;
- Feasibility and effectiveness of the proposed method were experimentally verified using a prototype of WSN. The conducted experiments have involved in comparison with state-of-the-art approaches.

The remainder of this paper is structured as follows. Section 2 reviews the most relevant previous works and discusses issues that have motivated our research. Section 3 presents details of the proposed method for data transmission reduction in event-detecting sensor networks. Experiments and their results are discussed in Section 4. Finally, conclusions are given in Section 5.

## 2. Related Works

Many works in the literature are devoted to reducing data transmission in WSNs. This section discusses the limitations of the existing transmission reduction methods suitable for application in WSN-based event detection systems. These methods fall under the following main categories: data aggregation and compression, adaptive sampling, dual prediction, and event-triggered transmission.

### 2.1. Compression

The compression methods allow us to reduce the size of the transmitted data. However, the decreased data size does not always translate into a lower number of transmitted packets and lower energy consumption. In general, compression is helpful when a considerable amount of data are collected (e.g., at the cluster head node, where data are received from many sensor nodes). For data transmission from a single sensor node, the application of compression techniques requires data buffering and can lead to significant delays in reporting important event-related data [17]. Thus, such an approach is not suitable for the detection of events in real time.

In related works, the compression approach was used for transmissions between cluster heads and sink nodes. Singh et al. [21] have considered a WSN where sensor nodes detect many events and report them to a cluster head. The cluster head recognizes important events based on data from several sensor nodes. The information about important events is then transmitted to a sink. In that scenario, the cluster head transmits compressed data to the sink, where the original data are reconstructed. Nagdive and Ingole [22] implemented the sequential lossless entropy compression (S-LEC) algorithm in WSN for the detection and localization of heterogeneous events. The S-LEC algorithm was used in their work to reduce the size of the data transmitted between cluster heads.

### 2.2. Aggregation

Data aggregation methods have similar limitations to data compression, as they assume that data from many sensor nodes must be merged. Aggregation usually involves a fusion of data from multiple sensor nodes at cluster heads (or intermediate nodes). Then, the aggregated data are transmitted to a sink. In [23], the in-network aggregation method was used for a cluster-based WSN which was designed to detect early fires. The cluster head in that WSN aggregates the CO, temperature, photoelectric, and ionization data obtained from different sensor nodes. After transmission, the sink uses the aggregated data as inputs of a neural network to recognize fire events. In [24], a multihop tree-based data aggregation framework was proposed for WSNs that monitor events in large areas. According to that approach, a multihop path is determined for transmitting event information to a sink via many intermediate nodes. The intermediate sensor nodes along the path aggregate the event data with information about other events in the monitored area to minimize the number of transmissions. Such an aggregation method can reduce the data transmissions only if there are many events detected in different locations simultaneously.

### 2.3. Adaptive Sampling

The adaptive sampling approach allows a sensor node to modify its sampling rate such that the sensor works with a high sampling rate to provide detailed data when observing an important event. When the sensor does not observe important events, a lower sampling rate is used. This method leads to a reduced amount of sampled, processed, and transmitted data [19]. The primary motivation for introducing the adaptive sampling mechanisms is that in some sensor nodes, a significant percentage of energy is consumed by the sensing module, and it is necessary to reduce this energy consumption for the purposes of WSN lifetime extension [25,26]. The adaptive sampling approach was used in WSN for detection of water leaks from pipelines [25]. This strategy uses two types of vibration and pressure sensors that have different precision and energy consumption levels. The sampling frequency is adjusted with the use of a wavelet-based adaptive thresholding scheme by taking into account the bandwidth of the acquired vibration signal. Measurements from energy-efficient low-precision vibration sensors are used in that method to adjust the sampling rate of the sensors having high precision and high energy consumption.

Adaptive sampling was also used for structural health monitoring and fire event monitoring. In this context, it was observed that in practice, the application of the adaptive sampling approach for event detection was challenging since the presence of a physical event is dynamic or unknown until after sampling [27,28]. Thus, this technique can make a WSN unable to capture dynamic short-term changes that correspond to the events of interest.

### 2.4. Dual Prediction

According to the dual prediction approach, the wireless nodes in WSN use a prediction model to compute estimates of sensors readings [18,28]. In this approach, predictions are simultaneously made by a sink and a sensor node. The sensor node needs the prediction to decide whether its current sensor readings have to be transmitted to the sink. More specifically, the sensor node sends its data readings to the sink if a difference between the prediction and real measured value is above a given threshold. The sink uses the predicted values to reconstruct missing data when the sensor node suppresses transmissions. When data transmission is performed, the sink substitutes its local predictions with the correct value received from the sensor node. Hence, measurements are transmitted to the sink only when the predictions are not sufficiently accurate. As a result, sensor nodes can avoid unnecessary transmissions, consume less energy, and extend their lifetime [29].

In [28], it was suggested that data reduction techniques based on dual prediction are more suitable than adaptive sampling for applications in event detection systems (e.g., intruder detection), where sudden variations of a physical variable may indicate the occurrence of an important event. However, the effectiveness of the dual prediction scheme in reducing data transmissions depends on the accuracy of the prediction model. Thus, extended dual prediction methods were presented in the literature to tackle the problem of eliminating erroneous sensor data (outliers) that lead to wrong predictions [30].

### 2.5. Event-Triggered Transmission

The event-triggered approach to data transmission in WSN assumes that data are sent from sensor nodes only when some specific event occurs. This means that after each data sampling, the sensor node verifies an event-triggering condition to decide whether or not to transmit the newly sampled data [1]. The event triggering condition can be verified using thresholds. Another approach is to detect events with the use of machine-learning techniques. The threshold-based methods include static and dynamic event-triggered schemes. Static schemes use fixed threshold values [31]. In the case of dynamic schemes, the thresholds are dynamically adjusted during the operation of WSN [32]. Different machine-learning algorithms were used in the literature to enable recognizing various events by sensor nodes. Support vector machine and decision tree were employed for occupancy detection [33]. A decision tree was also implemented in a wearable sensor node to detect the activities of house occupants [34]. In [35], a technique was proposed for leakage detection in pipelines with the use of the support vector machine, k-nearest neighbor algorithm, and the Gaussian mixture model. Another example is the application of a convolutional neural network for footstep detection [36].

The aforementioned event-triggered methods are suitable for data transmission reduction when detecting events based on local measurements performed by one sensor node. Other methods have been proposed for applications, where the events need to be detected based on data collected from neighboring sensor nodes [36,37]. However, those methods do not reduce the number of one-hop transmissions between sensor nodes in a neighborhood. A modified event-triggered approach was formulated in [20] as a domain reduction model. This method can be implemented only for threshold-based event detection.

## 3. Proposed Method

The proposed method was designed to reduce data transmissions between the neighboring sensor nodes that cooperate in detecting events. This method can be implemented together with periodic or adaptive sampling as well as with machine learning or threshold-based event detection algorithms. It significantly extends the concept of dual prediction by taking into account the usefulness of sensor readings for a given event detection task. In contrast to the dual prediction scheme, the proposed method does not use prediction error to decide whether the sensed data have to be transmitted. Instead, this method verifies whether the predicted data are sufficient for correctly recognizing the events of interest.

The objective of the considered wireless sensor network is to detect spatial events that result in divergences between readings of neighboring sensor nodes. The wireless sensor network is composed of parent nodes and child nodes. The child node reports its sensor readings to a parent node. The task of the parent node is to detect events based on both its own sensor readings and sensor readings delivered by the child node. When an event is detected, the parent node sends information to a base station. Formally, the event detection task, performed by the parent node, can be described as a binary function:

(1) $$e_{t}=event\left(s_{p,t},s_{c,t},E\right),$$

where $e_{t}=1$ if an event is detected and $e_{t}=0$ otherwise. Symbols $s_{p,t}$ and $s_{c,t}$ denote sensor readings registered at time step *t* by the parent node and child node, respectively. *E* stands for the event detection model, which is created (trained) in advance based on historical data. This model can take different forms, such as decision rules, decision tree, random forest, neural net, etc.

In order to reduce the data transmission, the child node sends its sensor readings $s_{c,t}$ to the parent node at selected time steps. For the remaining time steps, the parent node has to predict the sensor readings of the child node and detect events using the predicted values ${\hat{s}}_{c,t}$. Here, we use symbols $e_{t}$ and ${\hat{e}}_{t}$ to distinguish between the results $e_{t}$ of event detection from the complete, actual data ($s_{c,t}$) and results ${\hat{e}}_{t}$ that can be obtained with use of the predicted data (${\hat{s}}_{c,t}$), when transmission reduction is implemented:

(2) $${\hat{e}}_{t}=\left\{\begin{array}{cc} event(s_{p,t},s_{c,t},E), & tx_{t}=1\\ event(s_{p,t},{\hat{s}}_{c,t},E), & tx_{t}=0 \end{array}\right.,$$

where $tx_{t}$ is a binary variable, which determines whether data from child node are delivered to parent node at time *t* ($tx_{t}=1$ if data are transmitted and $tx_{t}=0$ in opposite situation).

For the above-discussed scenario of data transmission reduction, the fundamental problem is to decide when the child node has to send its current sensor readings to the parent node. Note that the number of data transmissions should be as low as possible, and at the same time, the transmitted data have to be sufficient for the correct detection of events. This task can be formulated as an optimization problem:

(3) $$\begin{array}{cc} \; & \min_{tx_{1}\cdots tx_{T}}\sum_{t=1}^{T}tx_{t}\\ \; & \mathrm{subject}\;\mathrm{to}\;\;{\hat{e}}_{t}=e_{t},\;t=1\cdots T \end{array}$$

Since we require the real-time operation of the event detection system, the decision variable $tx_{t}$ has to be determined at time step *t*, when the future sensor readings ($s_{p,t+1},\cdots ,s_{p,T}$ and $s_{c,t+1},\cdots ,s_{c,T}$) are unknown and previously taken decisions ($tx_{1},\cdots ,tx_{t-1}$) cannot be changed. Thus, the solution for problem (3) is given by the following formula:

(4) $$tx_{t}=\left\{\begin{array}{cc} 1, & event\left(s_{p,t},{\hat{s}}_{c,t},E\right)\neq e_{t}\\ 0, & \mathrm{else} \end{array}\right.,$$

which means that at a given time step (*t*), the data transmission should be performed by child node ($tx_{t}=1$) only if the event $e_{t}$ cannot be correctly recognized by the parent node based on the prediction results.

In practice, the solution (4) is not suitable for direct implementation as $e_{t}$ cannot be determined by sensor nodes without exchanging data. Specifically, a child node would need to receive $s_{p,t}$ form the parent node to determine $e_{t}$. Thus, data transmission would be necessary at each time step, which is clearly contrary to the objective of transmission reduction.

Therefore, a different approach was used in the proposed method. According to this approach, the child node takes into account possible values of $s_{p,t}$ and sends $s_{c,t}$ to the parent node if there is a possibility that the condition $event\left(s_{p,t},{\hat{s}}_{c,t},E\right)\neq e_{t}$ is satisfied. To this end, the child node finds the set ${\hat{S}}_{p,t}$, which includes possible sensor readings of the parent node. This set is determined based on model $M_{p}$, which is created using previously collected data. Then, the child node verifies whether the results of event detection, obtained for its actual and predicted sensor readings ($s_{c,t}$ and ${\hat{s}}_{c,t}$), are the same. This verification was made for all possible sensor readings of the parent node in ${\hat{S}}_{p,t}$. If the compared results of event detection are different for any of the elements in ${\hat{S}}_{p,t}$, then it is possible that event $e_{t}$ is incorrectly recognized when the parent node takes into account the predicted sensor readings of the child node. Thus, the approximate solution of the problem (3) is obtained as follows:

(5) $${\tilde{tx}}_{t}=\left\{\begin{array}{cc} 1, & \exists \;{\hat{s}}_{p,t}\in {\hat{S}}_{p,t}:event\left({\hat{s}}_{p,t},{\hat{s}}_{c,t},E\right)\neq event\left({\hat{s}}_{p,t},s_{c,t},E\right)\\ 0, & \mathrm{else} \end{array}\right.,$$

Obviously, this approach can give a sub-optimal solution of problem (3) as some unnecessary transmissions may be performed when the condition in (5) is not met for ${\hat{s}}_{p,t}=s_{p,t}$. However, it ensures that the constraint in (3) is fulfilled, which is of primary importance for correct event detection in the considered sensor network. This approach is motivated by the requirement that transmission reduction cannot change the results of event detection.

Details of the proposed transmission reduction method are presented in this section by pseudo-code. Operations of the parent sensor node are shown in Algorithm 1, while Algorithm 2 describes the operation of the child sensor node. These algorithms allow us to summarize the above-discussed concepts.

As shown in Algorithm 1 (line 9), the parent node stores the received sensor readings of the child node ($s_{c,t}$) and uses the stored data ($s_{c}$) for event detection in case the child node does not send its current data. This means that this algorithm implements the Naive prediction method [18] to predict the sensor readings of the child node that are denoted by symbol ${\hat{s}}_{c,t}$ in Equations (2)–(5). The choice of Naive prediction was motivated by the fact that this method was found to be effective in predicting a chaotic time series that correspond to unexpected events, such as accelerations registered for the sudden movement of an object [15].
**Algorithm 1** Operation of parent node1:**Inputs:**    event detection model *E*2:**Initialize:**    determine parameters *P* of prediction model $M_{p}$3:**for** each time step *t* **do**4:    collect data $s_{p,t}$ from own sensors5:    update parameters *P* of prediction model $M_{p}$6:    wait for data $s_{c,t}$ transmitted from child sensor node7:    **if** data $s_{c,t}$ received **then**8:        send acknowledgement with $s_{p,t}$ and *P* to child node9:        $s_{c}\leftarrow s_{c,t}$10:    **end if**11:    **if** $event(s_{p,t},s_{c},E)=true$ **then**12:        report event to sink13:    **end if**14:**end for**

**Algorithm 2** Operation of child node
1:
**Inputs:**
    event detection model *E*    parent data prediction model *M_p_*2:
**Initialize:**
    tx←13:**for** each time step *t* **do**4:    collect data $s_{c,t}$ from own sensors5:    **if** tx=0 **then**6:        predict possible data readings of parent node ${\hat{S}}_{p,t}\leftarrow prediction\left({\hat{S}}_{p,t-1},M_{p},\hat{P}\right)$7:        **if** ${\hat{s}}_{p}$ exists, such that ${\hat{s}}_{p}\in {\hat{S}}_{p,t}$ and $event\left({\hat{s}}_{p,t},{\hat{s}}_{c},E\right)\neq event\left({\hat{s}}_{p,t},s_{c,t},E\right)$ **then**8:           tx←19:        **end if**10:    **end if**11:    **if** tx=1 **then**12:        send $s_{c,t}$ to parent node13:        receive acknowledgement with $s_{p,t}$ and *P* from parent node14:        ${\hat{S}}_{p,t}\leftarrow \left\{s_{p,t}\right\}$15:        $\hat{P}\leftarrow P$16:        ${\hat{s}}_{c}\leftarrow s_{c,t}$17:        tx←018:    **end if**19:
**end for**



It should also be noted here that the parent node acknowledges the reception of messages from the child node (line 8 in Algorithm 1). In addition to ensuring the reliability of data transmission, acknowledgements are also used to inform the child node about the current sensor readings of parent nodes and the parameters of the prediction model $M_{p}$. As already mentioned, model $M_{p}$ is used by the child node to determine the possible sensor readings of the parent node.

In the simplest case, the variables $s_{p,t}$ and $s_{c,t}$ are single values (scalars) representing, e.g., readings collected by a node with only one simple sensor. When the network nodes are equipped with more complex sensors or algorithms, the variables mentioned above are vectors that can include many sensor readings or results of data processing. Thus, for the sake of generality, we use the following vector notation:

(6) $$s_{p,t}=\langle s_{p,t,i},i=1\cdots n\rangle ,\;s_{c,t}=\langle s_{c,t,i},i=1\cdots n\rangle .$$

In order to determine the set ${\hat{S}}_{p,t}$ of possible parent node readings, an interval of possible values is assigned to each component of $s_{p,t}$. This assumption leads to the formula:

(7) $${\hat{S}}_{p,t}=\langle \left[{\hat{S}}_{p,t,i}^{-},{\hat{S}}_{p,t,i}^{+}\right],i=1\cdots n\rangle ,$$

where the superscripts − and + indicate the lower and upper endpoint of the interval.

At each time step of the sensor network operation, the intervals in ${\hat{S}}_{p,t}$ are updated using the prediction function (line 6 in Algorithm 2) to calculate the new interval endpoints:

(8) $${\hat{S}}_{p,t,i}^{-}={\hat{S}}_{p,t-1,i}^{-}+{\hat{p}}_{i}^{-},\;{\hat{S}}_{p,t,i}^{+}={\hat{S}}_{p,t-1,i}^{+}+{\hat{p}}_{i}^{+},\;i=1\cdots n$$

Parameters ${\hat{p}}_{i}^{-}$ and ${\hat{p}}_{i}^{+}$ describe the possible range of difference between $s_{p,t-1,i}$ and $s_{p,t,i}$. Values of these parameters are evaluated by the parent node with the use of the algorithm for quantiles tracking in data streams [38]. Specifically, ${\hat{p}}_{i}^{+}$ corresponds to the *k*-th percentile of the positive values of difference $\left(s_{p,t,i}-s_{p,t-1,i}\right)$ and ${\hat{p}}_{i}^{-}$ is determined as the (100−k)-th percentile of the negative values of this difference between two successive measurements.

If the data transmission is executed at a given time step, the updated intervals are determined based on the actual $s_{p,t,i}$ values, reported by the parent node:

(9) $${\hat{S}}_{p,t,i}^{-}=s_{p,t,i},\;{\hat{S}}_{p,t,i}^{+}=s_{p,t,i},\;i=1\cdots n$$

**Figure 1:**
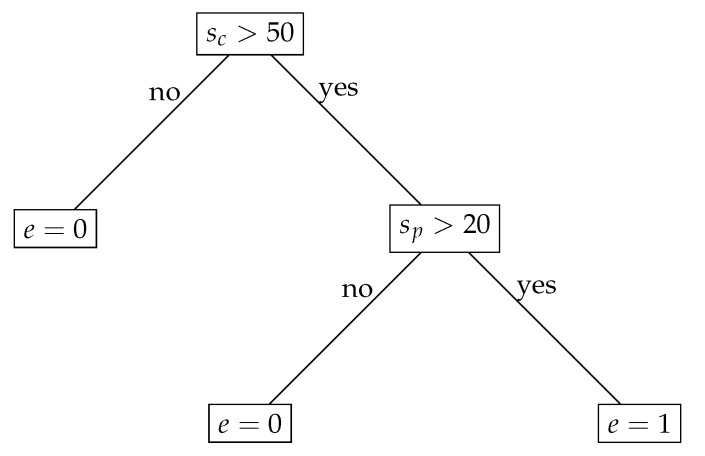
Decision tree.

After finding the set of possible sensor readings of the parent node, the child node has to decide whether its sensor readings must be sent to the parent node. This decision is taken in line 7 of Algorithm 2. Details of this operation are discussed here using a simple example of the event detection model *E* in the form of the decision tree (Figure 1). For illustrative purposes, it was assumed that both the parent and the child sensor nodes only monitor one parameter ($s_{p}$ and $s_{c}$). Moreover, to simplify notation, the indices *t* and *i* are ignored.

The decision tree from Figure 1 can be converted into the following set of decision rules:

1. if $s_{c}\leq 50$ then e=0,
2. if $s_{c}>50$ and $s_{p}\leq 20$ then e=0,
3. if $s_{c}>50$ and $s_{p}>20$ then e=1.

Let us assume that the interval of the possible parent node readings is ${\hat{S}}_{p,t}=\left[10,25\right]$, the predicted sensor reading of child node is ${\hat{s}}_{c}=25$, and the actual child node reading equals $s_{c,t}=60$.

For the above assumptions, we check whether $e=event({\hat{s}}_{p,t},{\hat{s}}_{c},E)$ can take values 0 and 1. To this end, $s_{c}$ in the decision rules is substituted by ${\hat{s}}_{c}=25$ and $s_{p}$ is substituted by ${\hat{s}}_{p}\in \left[10,25\right]$. In this case, we obtain e=0 from the first decision rule. The conditions in rules 2 and 3 are not satisfied.

Then, we check whether different event detection results (e=1) are possible when taking into account the current sensor reading of the child node. This means that we must verify whether $e=event({\hat{s}}_{p,t},s_{c,t},E)$ can take value 1. Thus, in the decision rules, $s_{c}$ is substituted by $s_{c,t}=60$ and $s_{p}$ is substituted by ${\hat{s}}_{p}\in \left[10,25\right]$. In this case, e=1 is possible under the condition that rule 3 is satisfied.

Based on the above analysis, we conclude that the results of event detection for $event({\hat{s}}_{p,t},{\hat{s}}_{c},E)$ and $event({\hat{s}}_{p,t},s_{c,t},E)$ can be different (e=0 in the first analyzed case and e=1 in the second case), which means that the actual sensor reading ($s_{c,t}$) has to be transmitted to the parent node (tx=1).

Let us consider another situation, when ${\hat{S}}_{p,t}=\left[0,15\right]$, ${\hat{s}}_{c}=25$, and $s_{c,t}=60$. In this case, we obtain $event({\hat{s}}_{p,t},{\hat{s}}_{c},E)=0$ from rule 1 and $event({\hat{s}}_{p,t},s_{c,t},E)=0$ from rule 2. For the remaining rules, the conditions are not met. It shows that the result of event detection, obtained for the predicted and for the actual readings of the child node, are the same. Thus, the data transmission is skipped (tx=0).

Finally, let us take into account the following assumptions: ${\hat{S}}_{p,t}=\left[10,25\right]$, ${\hat{s}}_{c}=100$, and $s_{c,t}=60$. In this case, we obtain $event({\hat{s}}_{p,t},{\hat{s}}_{c},E)=1$ from rule 3 and $event({\hat{s}}_{p,t},s_{c,t},E)=0$ from rule 2. Rule 1 cannot be satisfied in this case. Here, the result of event detection can be different due to rules 2 and 3. However, the data transmission is skipped (tx=0) because rules 2 and 3 cannot be satisfied for the same $s_{p}$ value, which means that the result of event detection will not change after reporting $s_{c,t}=60$ to the parent node.

From the above example, we can conclude that the sensor readings of the child node have to be transmitted (tx=1) if the set of decision rules for model *E* includes two rules $r_{1}$ and $r_{2}$, such that:

- The outcomes of $r_{1}$ and $r_{2}$ are different;
- The condition of $r_{1}$ may be satisfied for $s_{c}=s_{c,t}$;
- The condition of $r_{2}$ may be satisfied for $s_{c}={\hat{s}}_{c}$;
- $s_{p}\in {\hat{S}}_{p,t}$ exists for which conditions of both $r_{1}$ and $r_{2}$ may be satisfied.

In the opposite situation, data transmission is not performed (tx=0).

It should be noted here that the approach presented in the above example can be implemented in more complex scenarios. If a more elaborate model is used for event detection, then a larger set of decision rules must be analyzed. The decision rules can be directly extracted from multiple trees forming the random forest model [39]. Appropriate algorithms are also available in the literature for the decision tree, and the decision rules’ extraction from neural networks [40] as well as from support vector machines [41]. The presented approach is also applicable when the sensor nodes collect more than one attribute, as the decision rules can account for a larger set of attributes.

The sensor nodes have to execute their tasks in a limited amount of time. Thus, it is important to analyze the computational complexity of Algorithms 1 and 2. We should especially consider the body of the for loop, which contains operations performed during each time step (*t*). The most computationally expensive process in these algorithms is that of event detection, i.e., the evaluation of the event function. As discussed above, we can assume that the event detection model *E* is a set of decision rules. Thus, the dominant operation is the verification of a condition for decision rule. Let *m* denote the number of decision rules in *E*. The parent node verifies the conditions *m* times as the event function is used once during a time step (line 11 in Algorithm 1). In the case of the child node, the number of dominant operations is 2m because each decision rule has to be applied two times to check the condition in line 7 of Algorithm 2, as described above for the example in Figure 1. This means that both algorithms (Algorithms 1 and 2) have linear time complexity O(m).

## 4. Experiments and Results

The objective of the conducted experiments was to test the proposed method on a real-world working event detection system. Our method was experimentally verified in a prototype WSN for cargo monitoring during transportation. The monitored events have included the movements of cargo boxes that are symptoms of improperly secured load in a vehicle. The objective of this monitoring system was the recognition of threats of possible damage to cargo and inform the users (e.g., driver, dispatcher, sender). The construction of the system was inspired by previous works on cargo monitoring in transportation and logistics systems [13,42,43]. During experiments, the accuracy of event detection was analyzed, and measurements were performed to determine the energy consumption of sensor nodes and to evaluate their lifetime. The results obtained for the proposed method were compared with those of the state-of-the-art dual prediction [18,44] and event-triggered transmission [33] methods. Moreover, in our research, state-of-the-art methods were implemented using different prediction and event recognition algorithms.

### 4.1. Experimental Testbed

**Figure 2:**
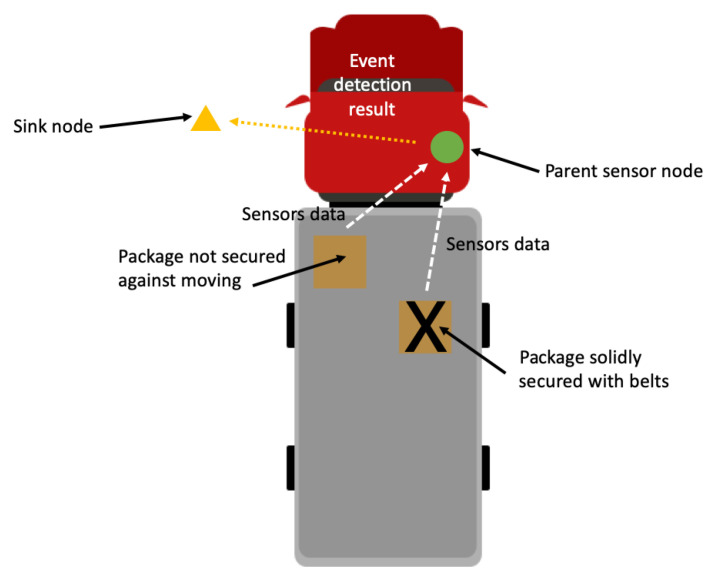
Schematic view of WSN for cargo monitoring.

A schema of the considered cargo monitoring system is shown in Figure 2. For the purpose of the conducted experiments, a prototype of the WSN network was built. The experimental WSN consisted of four nodes. Three of them were attached to cargo boxes and used as child nodes. The parent sensor node was placed at the vehicle body. This node was responsible for collecting data from child nodes and recognizing the events of interest.

Each sensor node of the considered WSN contains a microcontroller, accelerometer, gyroscope, and a communication module. As discussed in [45], the accelerometer and gyroscope sensors are useful to identify the forces acting on a vehicle and the cargo. In addition, the sensor nodes were equipped with a module responsible for measuring energy consumption. The measurement of energy consumption was performed by using the LTC4150 Coulomb counter. This module constantly monitors the current a node consumes, integrates it, and gives a pulse each time a given amount of amp-hours are used. The LTC4150 module generates an impulse on an INT pin each time the sensor node absorbs 0.1707 mAh. It was assumed that all child sensor nodes have the same battery capacity of 3500 mWh. Moreover, the LTC4150 module has a polarity output (POL). The signal on the POL output indicates the direction of the current flow. A low state on this output during the INT impulse means that the battery is discharging.

The data transmitted from a child node to the parent node consist of the accelerometer reading (7 bytes) and gyroscope reading (7 bytes). The response of the parent node includes its sensor readings (14 bytes) and updated parameters P of the prediction model (28 bytes). In the case when the transmission reduction is not performed, the child node sends one message per second.

Lifetime of WSN is defined in the literature by taking into account different situations [46]: first node discharge (FND—first node dead), last node discharge (LND—last node dead) and half of the available nodes discharge (HND—half node dead). For our cargo monitoring system, all the battery-powered sensor nodes are necessary to detect the events of interest. In this study, it was assumed that the minimum number of child nodes is one, which corresponds to one monitored package. Thus, the term lifetime refers to the time of the WSN operation before the death of the first sensor node. The death of the sensor node was detected during the experiments each time the sensor node had consumed a predetermined amount of energy (3500 mWh).

The prototype sensor nodes use ARM microcontrollers (STM32F103C8T6) which allow us to switch off individual modules that are not used and reduce energy consumption. This hardware platform also offers a DMA controller, which has enabled the implementation of time-effective data collection and processing procedures.

The wireless communication of sensor nodes was based on Zigbee technology (xBee S2C module). The microcontroller was connected with the communication module through the Universal Asynchronous Receiver Transmitter (UART) interface. The communication module is put in sleep mode when the data transmission is not performed. Additionally, this module sends special data frames to inform the system about exceptional situations (e.g., communication errors).

The sensors’ subsystem of the sensor nodes was based on the GY-91 module. This 10 DoF (degrees of freedom) module contains a combination of single-chip MPU-9250 with a built-in three-axis gyroscope, three-axis accelerometer, digital compass, and BMP280 improved barometric pressure sensor. As discussed earlier in this section, the data from the accelerometer and gyroscope were used to detect the movements of cargo boxes in a vehicle.

The STM32 microcontroller in our prototype has rich peripherals, high computing power, and a DMA mechanism, which enable fast and energy-efficient communication with the sensing and wireless transmission modules. The aforementioned features of the hardware platform are essential for our research studies devoted to various applications of WSNs (e.g., human activity recognition [14] and road traffic monitoring [47]). The cost of the microcontroller does not exceed USD 5. The communication module in our prototype was based on ZigBee technology; however, cheaper alternatives are available for wireless end-point communication at the cost of USD 2. The cost of the GY-91 sensing module is USD 8.

### 4.2. Results and Discussion

A dataset for our experiments was collected using the WSN prototype in a car traveling on urban roads in Sosnowiec, Poland. The routes were selected to cover many crossroads, roundabouts, and curves. When collecting data, we avoided traffic congestion and peak hours. Thus, the movements of unsecured packages in the car were very frequent. The data collection was conducted for three working days. A passenger in the car was controlling the situation by selectively holding and releasing packages for short time periods so that the released package was moving. We marked the events by registering the time when a given package was moving inside the car. The dataset includes a total number of 6909 events.

The first experiments were conducted to verify the possibility of detecting the events of interest and comparing the accuracy of cargo movement detection for various machine learning classification algorithms. The compared machine learning algorithms include: k-nearest neighbors (kNN) [48], multilayer perceptron (MLP)—which is a popular example of neural network [49]—probabilistic neural network (PNN) [50], random tree (RT) [51], and random forest (RF) [52]. All tests of the above-listed algorithms were performed using their implementations available in the Konstanz Information Miner (KNIME) and WEKA package. During the experiments, 60% of the collected data were used for training and 40% for testing.

**Figure 3:**
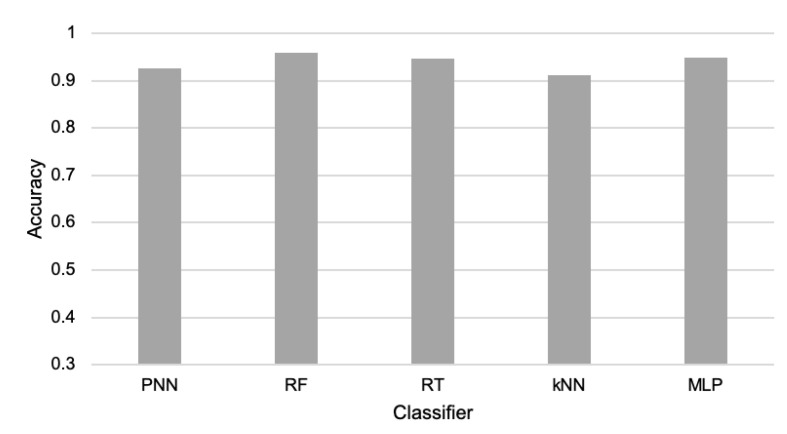
Accuracy of event detection for compared machine learning algorithms.

During the first part of the experiments, the reduction in data transmission was not performed; thus, all available data were taken into account. It means that the events were detected based on sensor readings from both the child and parent node. The results presented in Figure 3 show that the collected data allow all the considered algorithms to detect the events of interest with a high level of accuracy. These results confirm that the elaborated WSN is useful for cargo monitoring during transportation. The detection accuracy obtained for particular machine learning algorithms was as follows: PNN achieved 92.5%; RF 95.9%; RT 94.6%; kNN 91.2%; and MLP 94.9%. The differences in those results are quite low, especially for RF, RT, and MLP. Thus, all three of these algorithms are candidates for practical applications. However, the RF algorithm, which achieved the highest detection accuracy, was selected for further experiments on data transmission reduction.

The RF algorithm can be considered as an extended version of the RT method where a collection of decision trees is built. The RF algorithm creates decision trees using a random procedure. The construction of a decision tree involves the greedy selection of the best split point from the dataset at each step. By creating multiple trees with different samples of the training dataset, the RF algorithm introduces different views of the detection problem. This algorithm uses the majority voting scheme to determine the final classification result. As shown in the literature, the RF algorithm can be successfully implemented in embedded systems [53].

**Figure 4:**
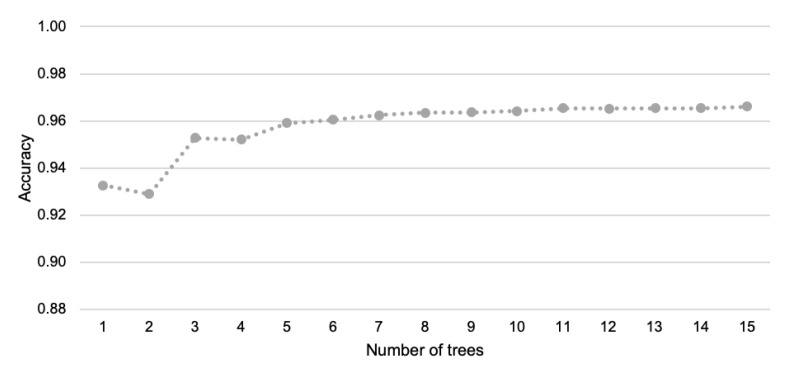
Accuracy of event detection for different numbers of trees.

Figure 4 shows the accuracy of event detection with the use of the RF algorithm for different numbers of trees. Based on this chart, it can be observed that when the number of trees is increased, the impact on recognition accuracy is not significant. This effect is especially visible when the number of trees is greater than 10 (the dotted line is almost flat). It should be noted here that the lower number of trees leads to the lower computational complexity of the detection procedure, which is desirable from the perspective of the implementation in sensor nodes. Thus, the RF algorithm was used in our experiments with ten trees.

**Figure 5:**
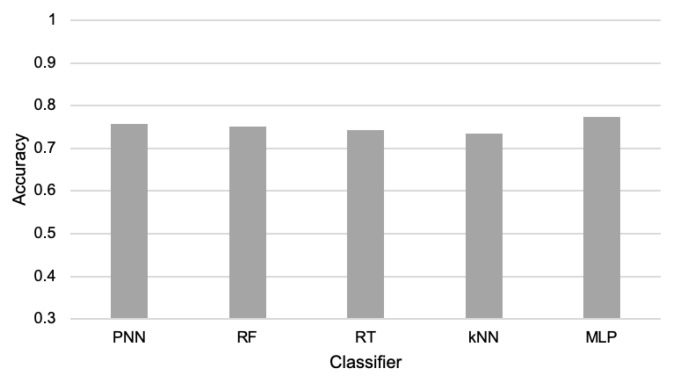
Accuracy of the parcel movement recognition for event-triggered approach.

The second part of the experiments was devoted to the evaluation of the event-triggered approach [33]. In this case, the child nodes detect events based on their own sensor readings. The amount of data transfers is reduced in this method since the child nodes send information to the parent node only when they detect movements of the packages. In order to detect the events, the same five machine learning algorithms were used, as in the previous experiments. However, the sensor readings of the parent node were excluded from the training and testing datasets because when using the state-of-the-art event-triggered approach, the nodes do not exchange their sensor readings. Thus, the child sensor node has to detect events based on locally collected data. The results obtained for the event-triggered approach are presented in Figure 5. When comparing the charts in Figure 3 and Figure 5, it can be observed that for all considered algorithms, the accuracy of event detection is significantly decreased when using the event-triggered transmission. These results show that the events cannot be accurately recognized by the child node only. Hence, in the proposed method, the child and parent nodes cooperate in detecting events. Due to the low detection accuracy (between 73% and 77%), we found that the event-triggered method was not applicable for the cargo monitoring system. Another disadvantage of this method was the lower network lifetime in comparison to the proposed method (120 h vs. 125 h).

In the next part of the experiments, the effectiveness of the proposed transmission reduction method was compared with that of the dual prediction strategy. Two versions of the dual prediction method use different prediction models—the naive model [44] and the neural network model [18]—were taken into account.

**Figure 6:**
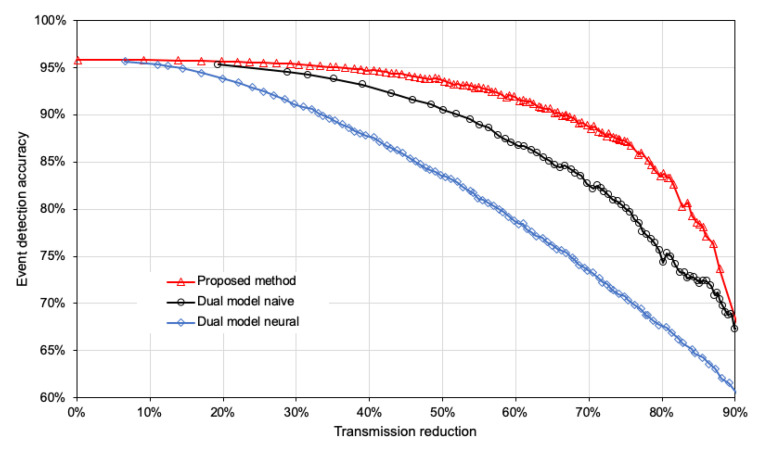
Event detection accuracy and transmission reduction for compared methods.

The results presented in Figure 6 show the dependency between event detection accuracy and transmission reduction. For all compared methods, the accuracy of event detection decreases when increasing the percentage of reduced transmissions. However, this decrease in accuracy is slowest in the case of the proposed method. Let us assume that we can accept an accuracy decrease by 10% as a cost of reducing data transmissions. Then, the proposed method allows us to eliminate almost 79% of data transmissions, while state-of-the-art methods achieve a transmission reduction of 65.3% for the naive model and 46.9% for the neural model.

**Figure 7:**
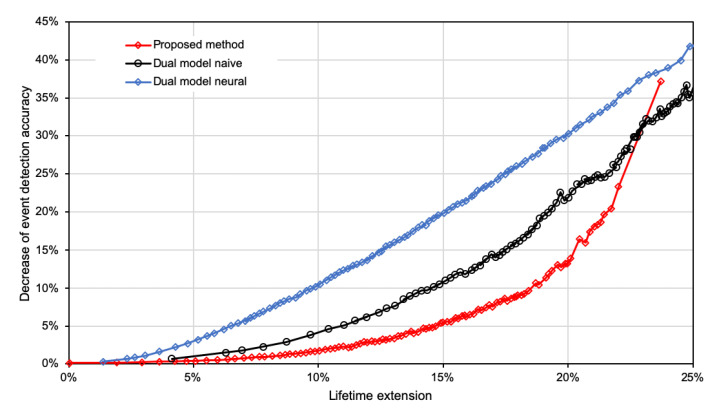
Impact of increasing data suppression on accuracy of event detection for compared methods.

The impact of the WSN lifetime extension on the decrease in detection accuracy is presented in Figure 7. It can be observed in these results that the state-of-the-art methods lead to a larger decrease in the detection accuracy in comparison to the proposed approach for a wide range of lifetime extensions. Thus, the trade-off between lifetime extension and accuracy decrease is more beneficial for the proposed solution. For instance, if we need to increase the network lifetime by 15%, then in the case of the proposed method, the accuracy is decreased by 5.6%. Still, the state-of-the-art methods decrease the accuracy level by 11% for the naive model and 20.3% for the neural model.

**Figure 8:**
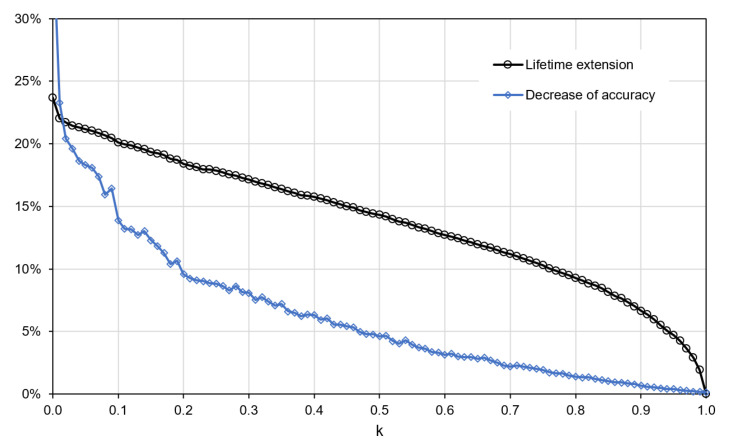
Impact of parameter *k* on lifetime extension and decrease in event detection accuracy for the proposed method.

It should be noted here that the particular data points on the charts in Figure 6, Figure 7 and Figure 8 correspond to the different settings of the transmission reduction algorithms. In the case of the state-of-the-art dual prediction algorithms, the threshold value was changed to collect these results. As it already explained in Section 2, the threshold determines how large of a prediction error will be accepted without transmitting the actual sensor readings. In the case of the proposed method, the results were collected for different values of the parameter *k*, which determines the percentiles taken into account when updating the interval of possible sensor readings in accordance with Equation (8). The impact of parameter *k* on the extension of the WSN lifetime and decrease in event detection accuracy is presented in Figure 8. For higher values of *k*, the interval of possible sensor readings is updated by taking into account less frequent, larger changes of the sensor readings observed in the past. Thus, the updated interval is wider and takes into account a larger set of possible changes that can occur in sensor readings between successive time steps of the sensor node operation. As a result, a lower decrease in event detection accuracy is observed for higher values of *k* because there is lower probability that the actual sensor reading will be outside the expected interval. The wider interval of possible sensor readings also means a higher probability that the condition, which determines the decision about transmitting data (line 7 in Algorithm 2) will be fulfilled. Thus, the number of transmission increases, and the WSN lifetime decreases with the increase in parameter *k*.

**Figure 9:**
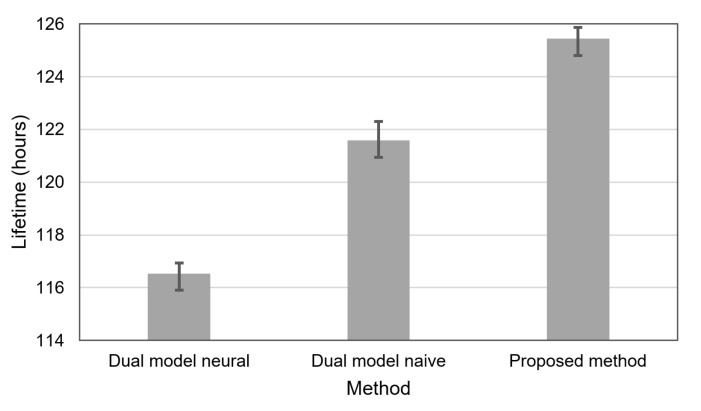
WSN lifetime for the compared methods in the case of 90% event detection accuracy.

Figure 9 shows the network lifetime that can be achieved for the compared methods when the accuracy of event detection equals 90%. The dual prediction method with the neural model allows the WSN to operate for 116 hours on average. In a situation when the dual prediction is applied with the naive model, the WSN average lifetime reaches 121 h. The longest average lifetime of 125 h was observed for the proposed method.

**Figure 10:**
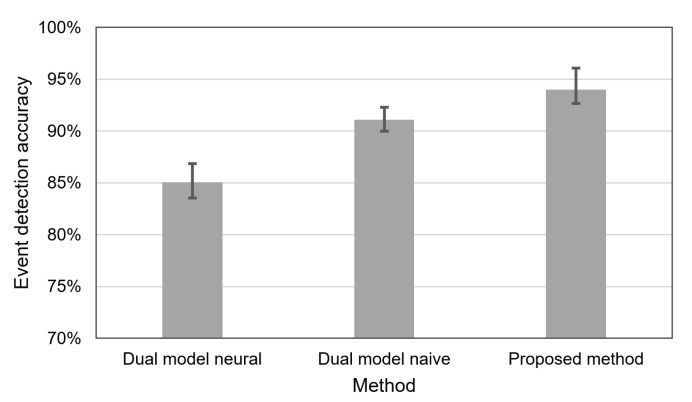
Accuracy of event detection for the compared methods when the WSN lifetime equals 120 h.

Figure 10 compares the accuracy of event detection, which was obtained in a situation when the lifetime of WSN was equal to 120 h. In this case, the dual prediction based on the neural model allows us to detect the events of interest with an average accuracy of 85.1%. When using the dual prediction with the naive model, we achieved the average accuracy of 91.1%. The highest event detection accuracy (94% on average) was observed for the proposed method. It should be noted that the columns in Figure 9 and Figure 10 correspond to average results, while error bars show minimal and maximal values obtained for 10 runs of the experiment. The better result of the naive approach in comparison with the dual prediction based on the neural model is a consequence of the fact that the neural network is less accurate in predicting the sensor readings. The best results were obtained for the proposed method because this method can better fit the data transmissions to the needs of event detection by taking into account the algorithm used for event detection. In contrast, state-of-the-art methods are agnostic with respect to the event detection algorithm.

**Figure 11:**
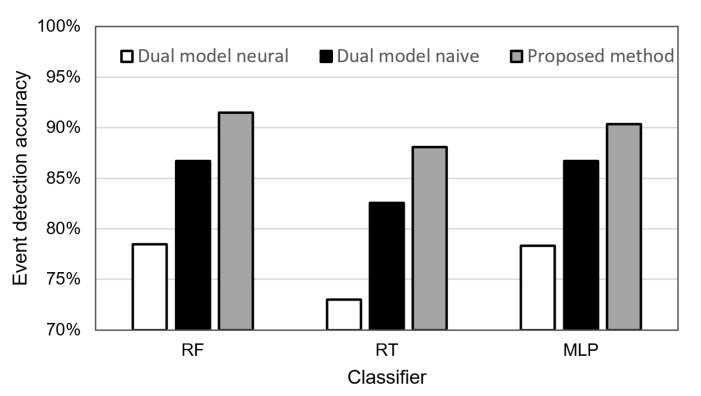
Accuracy of event detection with the use of different classifiers for the compared transmission reduction methods.

As it already explained earlier in this section, we applied the RF algorithm to detect events in our experiments since this classifier achieved the best results during the preliminary tests (see Figure 3). Additional experiments were performed to compare the performance of RF with RT and MLP algorithms when detecting events with the use of the reduced data. For the considered scenarios, 60% of the data transmissions were reduced. The results of these experiments are presented in Figure 11. They show that the RF classifier also has a higher event detection accuracy than RT and MLP for reduced data. The superiority of the RF classifier was observed for all the three considered transmission reduction methods.

**Figure 12:**
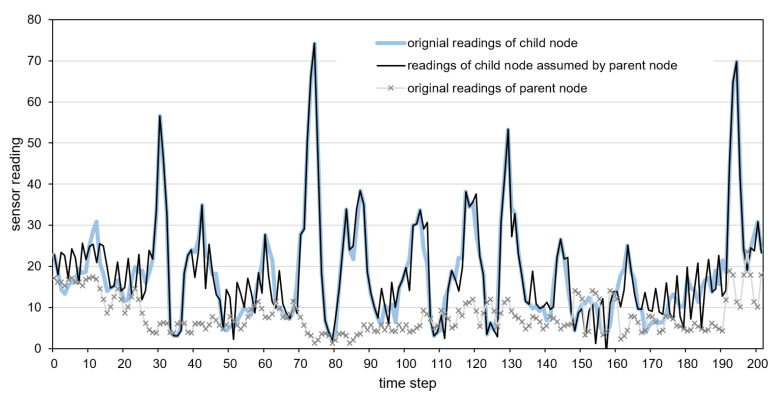
Sensor readings of the child node assumed by the parent node for the neural model.

**Figure 13:**
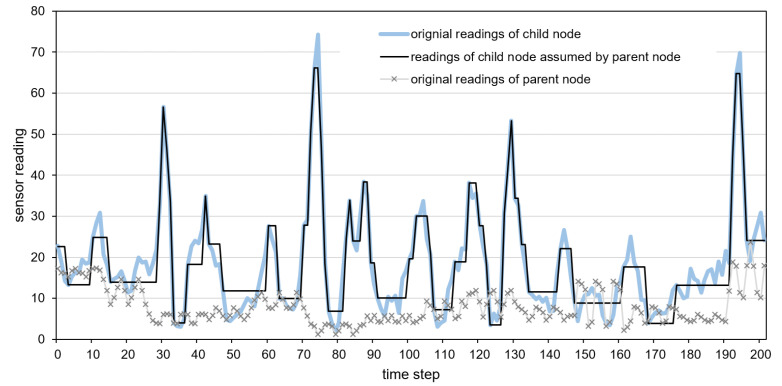
Sensor readings of the child node assumed by the parent node for the naive model.

**Figure 14:**
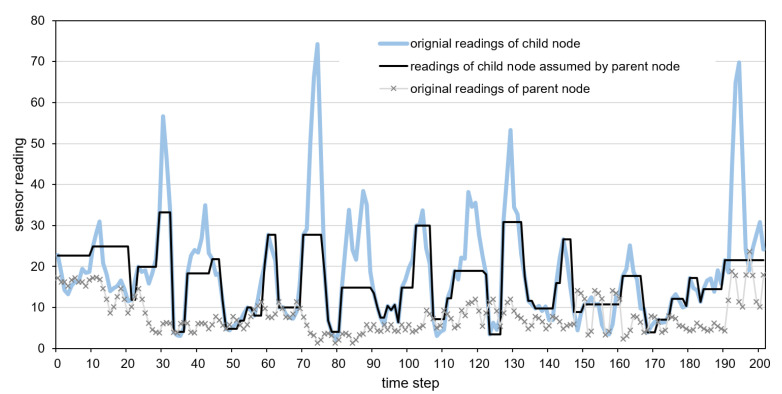
Sensor readings of the child node assumed by the parent node for the proposed method.

Detailed examples of data transmission reduction are presented in Figure 12, Figure 13 and Figure 14. These examples include the original time series of gyroscope readings registered by child and parent nodes and the time series of child nodes reconstructed by the parent node after reducing data transmissions. The parent sensor node was attached to the vehicle’s body, and the child node was placed inside a cargo box. The gyroscope readings shown in Figure 12, Figure 13 and Figure 14 were collected when traveling with the incorrectly secured cargo box that moves inside the vehicle. Therefore, the readings collected by the child node and parent node significantly differ. These differences allow us to recognize the events of interest, i.e., the movements of the cargo box. The reconstructed time series, which represents the sensor readings of the child node assumed by the parent node is depicted by the dark solid line. In Figure 13, the horizontal line segments correspond to the sensor readings predicted by the parent node during the periods in which data transmissions are not performed. In the case of the neural model, the predictions are visible in Figure 12 as the segments with high-frequency oscillations (e.g., between time steps 170 and 190).

When analyzing the presented examples, it can be observed that in the case of the dual prediction methods (Figure 12 and Figure 13), for both low and the high values, the sensor readings of the child node assumed by the parent node (dark solid line) are close to the original readings of the child node (light solid line). In contrast, when using the proposed method (Figure 14), the reconstructed time series of the sensor readings (dark solid line) is close to the original time series (light solid line) only for the low values. The proposed method does not report the high values of sensor readings (peaks) because, in the considered event detection algorithm, the child sensor reading may influence event detection if it is close to the parent sensor reading (dotted line). Thus, the detection results obtained without transmitting the high values of the child sensor readings (based on previously transmitted data) are the same as those obtained using the original sensor readings of the child node.

## 5. Conclusions and Future Work

The method presented in this paper reduces the number of data transmissions between neighboring sensor nodes that detect spatial events based on shared sensor readings. As a result of transmission reduction, the lifetime of WSN is prolonged. Moreover, the lower number of data transmissions leads to a reduced probability of collisions and delays in transmitting the data from sensor nodes.

In this study, the proposed method was applied in cargo monitoring systems to detect the movements of unsecured cargo boxes during transportation. Such a practical application example has enabled the demonstration of the advantages of our method. However, this method can be easily adapted for applications in various event detection systems.

The experiments revealed that the presented approach allows us to detect the events of interest with an accuracy of 90% when 65% of data transmissions between sensor nodes are eliminated. Based on the experimental evaluation results, it can be concluded that the introduced method is significantly more effective in reducing data transmissions and prolonging the lifetime of WSN than the state-of-the-art event-triggered transmission and dual prediction methods.

According to the proposed method, the child sensor node has to determine a set of possible sensor readings of the parent node to decide whether data transmission can be skipped. The set of possible sensor readings is determined with the use of a percentile tracking algorithm and represented by intervals. This means that all sensor readings in the interval are considered equally possible. Future research directions include extending this approach to distinguish between more and less possible sensor readings by using fuzzy sets and probabilistic models.

## Data Availability

Dataset analyzed in this study is publicly available after contact with the corresponding author: Bartłomiej Płaczek (bartlomiej.placzek@us.edu.pl).
